# Biomimetic Nanoscale Materials for Skin Cancer Therapy and Detection

**DOI:** 10.1155/2022/2961996

**Published:** 2022-04-07

**Authors:** Hamza Abu Owida

**Affiliations:** Medical Engineering Department, Faculty of Engineering, Al-Ahliyya Amman University, Amman 19328, Jordan

## Abstract

Skin cancer has developed as one of the most common types of cancer in the world, with a significant impact on public health impact and the economy. Nanotechnology methods for cancer treatment are appealing since they allow for the effective transport of medicines and other biologically active substances to specific tissues while minimizing harmful consequences. It is one of the most significant fields of research for treating skin cancer. Various nanomaterials have been employed in skin cancer therapy. The current review will summarize numerous methods of treating and diagnosing skin cancer in the earliest stages. There are numerous skin cancer indicators available for the prompt diagnosis of this type of disease. Traditional approaches to skin cancer diagnosis are explored, as are their shortcomings. Electrochemical and optical biosensors for skin cancer diagnosis and management were also discussed. Finally, various difficulties concerning the cost and ease of use of innovative methods should be addressed and overcome.

## 1. Introduction

Cancer is a phenomenon that strikes fear and anxiety when talked about; however, skin cancer has been the most underrated of all types of cancer [1]; nevertheless, this type of cancer has drawn the attention of scientists and researchers lately due to its high increase in skin cancer cases in the past 20 years; moreover, the attraction was triggered when it was noticed that there was a 300% increase in skin cancer cases dating from 1994 to 2014 [2].

There are several types of skin cancer, including keratinocytes skin cancer, melanoma skin cancer, and nonmelanoma skin cancer, as well as other skin cancers; for example, cutaneous lymphoma, Kaposi's sarcoma, and Merkel cell carcinoma are extremely uncommon to find [3]. In essence, those numbers concerning the increase in skin cancer cases have had a major impact on the health sector and are rather overwhelming globally [4].

Therefore, an immense amount of research has been conducted to find treatment for this type of cancer and to find ways and methods to use modern technology to ensure that the treatment is not only successful but also efficient [5]. Nevertheless, there are several types of treatment that are being used nowadays to cure skin cancer, and there are three main types of treatment using surgery, namely, cryosurgery, which involves freezing the cancer skin surface and extracting it [6]. Using a microscope, a small sample of the excised affected skin is used to examine the specimen for tumorous cells in a different type of surgery known as Mohs surgery [7]. In addition, he used a treatment method called shave excision, which is a straightforward procedure that involves removing tumor skin cells with a blade and then performing curettage and desiccation to remove the tumor's exposed skin with a curette [8]. During the last few decades, many recent years, many methodologies have been developed in skin cancer detection; one of the methods is the computer-aided diagnosis in the early stages which plays a vital role in determining the probability of getting cured [9, 10]. Murugan et al. proposed a support vector machine and *k* Nearest Neighbour classifier for melanoma detecting and identification of the melanocytes in the area of the epidermis [11]. Vijayalakshmi presented a completely automated system of convolutional neural networks and support vector machines to determine the accurate prediction of skin cancer and also to classify the skin cancer as malignant or nonmalignant melanoma [12]. Vocaturo et al. presented an application of a multiple-instance learning approach referring to the detection of melanoma by applying multiple-instance learning approaches to discriminate melanoma from dysplastic nevi and outline an even more complex challenge related to the classification of dysplastic nevi from common ones which could be the basis of more sophisticated tools useful for detecting skin cancer [13].

Technology nowadays is impressively advanced and has a broad spectrum to be implemented in many sectors and industries to increase reliability and efficiency [14]; scientists and researchers are finding ways to use this technology such as nanosystems to aid in drug delivery and enhance the therapeutic methods to cure patients with skin cancer; these systems that involve nanotechnology can be used in already present therapeutic methods such as chemotherapy [15, 16]; the efficiency of such methods can be increased leading to smaller doses and limiting them with increased effectivity [17]. The benefits of using nanotechnology to treat cancer and, in this case, skin cancer are numerous; however, researchers focus on the disadvantages, such as effective delivery and efficiency; additionally, it has proven to be multidrug-resistant and specific in its area of action [18]. Moreover, using this method has also been shown to diagnose the presence or absence of skin cancer; in many cases, underdiagnosis was a major issue in detecting the presence of skin cancer; nevertheless, the use of nanosystems has proven to tackle this issue of under diagnoses [19]. This method is of nanosystems and nanotechnology has evolved some spectacular nanostructured materials; examples of the materials are nanotubes, quantum dots, liposomes, nanomicelles, nanospheres, magnetic nanoparticles, nanostructured lipid carriers, and solid lipid nanoparticles.

## 2. Nanomaterials for Skin Cancer Therapy

There are many ways and methods for treating skin melanoma; the pharmaceutical industry is flooded with many types of treatments that have proven to be effective to a certain extent [2]. However, the barrier to which the drugs' effectiveness is limited comes from biological factors, such as the toxicity of active pharmaceutical ingredients by the liver. Moreover, applying such active pharmaceutical ingredients causes regular use of fast-depleting pharmacological ingredients which prevents large fluctuations in plasma concentrations [20].

The materials can eradicate those barriers and increase the efficiency of the drug activity to cure skin diseases and melanoma; moreover, there are several materials that have been constructed to aid with drug delivery for skin cancer patients such as dendrimers, liposomes, carbon derived nanoparticles, and protein-based and inorganic nanoformulation [21, 22].

Researchers' aim is to illuminate side effects caused by drugs or active pharmaceutical ingredients. The way to approach this goal is to use nanomaterials through specific targeting. This will allow the active pharmaceutical ingredients to be more efficient with limited doses, therefore, increasing the effectiveness and reducing toxicity. It has been used by physicians and dermatologists [23].

On many occasions in their use, they have proven to be highly effective and efficient with low levels of toxicity. Future studies will show more thorough results in curing other types of skin diseases with the aspect of understanding the correct amount of active pharmaceutical ingredients that can be delivered accurately [24, 25].

### 2.1. Lipid Nanoparticles

Lipid nanoparticles are mainly used as drug delivery vehicles due to their outstanding properties in drug delivery and their high biocompatibility, especially for skin cells [26]. There are various types of lipid nanoparticles, namely, nanostructured lipid cargo, cubosomes, nanodispersion, and niosomes.1 [27].

**Figure 1:**
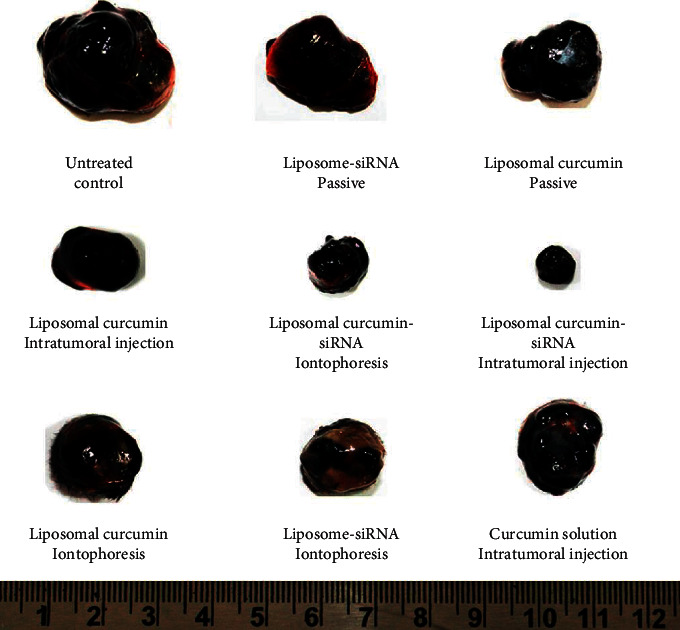
Tumors extracted from tumor-bearing mice after treatment with various formulations. All of the photos were of tumors isolated from five separate animals in a therapy group.

In every method, there are advantages and disadvantages. However, in this case, the advantages have a bigger margin due to the properties such as occlusive properties, alteration in release patterns, fewer side effects, and enhanced skin perforation [28]. Jain et al. stated that, in antiandrogens, especially RU 68841 myristate, strong lipid nanoparticles are converted to improve the proliferation and transfer of potent drug compounds. Liposomal descriptions of cyclosporine enhanced hair duplication in mice and demonstrated an important mechanism for fine-tuning alopecia aerate in humans, the author marked by the improved transfer of minoxidil depicted in nonpartisan liposomes into hair follicles compared to various systems. Various medications such as finasteride treat alopecia androgenetic [29]. Jose et al. focused on curcumin-mediated supplementation and hostility to STAT3 siRNA using cationic liposomes charged against skin melanoma. Curcumin or liposomal STAT3 siRNA can be packaged in liposomes to stop the growth of cancer because it is based on the weight of cancer. Liposomes are also used to measure epitome proficiency, zeta strength, molecular size, and how well they can be made. The cell line focuses on the occurrence of B16F10 observed in mouse melanoma cells showing that the similarity of both drugs inhibited the growth of disease cells compared to different data (Figure 1). Fine curosin-packed liposomes can penetrate the skin to the depth shown after using the iontophoresis strategy; moreover, in vivo studies were led to a model of melanoma skin disease in mice [30].

Caddeo et al. examined the production of liposomes, a combination of common polyphenols, which include resveratrol and quercetin. Polyphenols, when synthesized in liposomes, indicate higher cell proliferation than a single specialist. This review brings higher ROS roaming ability to fibroblast. The concentration of polyphenols accumulated in liposomes is estimated in the mouse model of skin removal. The active liposomal organization promotes inhibition of leukocyte infiltration and edema, which significantly improves tissue breakdown. As a result, the corresponding review considered that polyphenol embodiment into liposomes helps in the treatment of oxidative stress or stiffness associated with threatening skin lesions [31]. Skin fullness is focused on the results showing that liposomes iontophoresis is less susceptible to double 5-FU penetration into the epidermis compared with the same treatment with control liposomes controlled in the standing group. In vivo, subcutaneous administration of untreated liposomes reduces the risk of cancer reaching or occurring by more than 60 percent compared to poor control. Half the reduction in cancer capacity equated to control and 5-FU regimens that control the therapy of liposomes. These reviews show that immunoliposomes, which are made up of 5-FU skin organization and iontophoresis, are the best treatment for SCC [32].

### 2.2. Carbon Nanotubes

For the treatment of harmful skin growth, it is highly recommended to use carbon nanotubes (CNTs) for reasons such as their high stability and their high cytoprotective properties and their strong impacts on oxidants [33]. Ding et al. explore the meaning of harmful effects on the climate and the individual by nanotechnology application. Dermal fibroblast cell populations were introduced in multiwall CNTs to assess the total number of genomic articulation tests and phenotypic measurements developed in human skin. The findings show that the silent cells in the cytotoxic components of the two types of nanotubes begin to record the cell cycle that promotes decomposition. Furthermore, exposing these nanotubes regenerates the properties associated with cell transport, digestion, cell guidance, and so on. Microarray promoter experiments have revealed that the collapse of ERK-MAPK and interferon are fundamental components of signaling and participatory signaling pathways. The exposure of nanotubes revealed additional friendliness [34]. Hasebi and Hesabi are investigating the development of stable anticancer drugs and CNTs. The nanotube is made up of two compounds, aminolevulinic corrosive and tretinoin, which have been tested for sensitivity to various compounds. The results showed that the nanotube-aminolevulinic corrosive tretinoin was more stable than the nanotube-aminolevulinic corrosive tretinoin [35]. Moon et al. evaluated photothermally treatment using nanomaterials as an adequate disease-focused process. This work demonstrated the in vivo elimination of harmful growth by focusing on single-wall CNTs and NIR light. The heat-treated mice showed a complete remission of cancer without a long-term recurrence phase. As photothermally specialized materials, single-wall carbon nanotubes are a formidable and efficient competitor.The vast majority of lemon single-wall NTs were released from mice in about 2 months. The results demonstrated that single-wall CNTs complement each other as an active photothermally specialist and a platform for future growth risk [36]. Sahoo et al. created multiwalled carbon nanotubes and graphene oxide acting with biocompatible performance as well as hydrophilic packaging and concentrating enemy CPT camptothecin for malformation. CNT-PVA and GO-PVA were synthesized by pi-pi associations and demonstrated the potential to kill the breast and the malignant growth cells of the skin [37].

### 2.3. Gold Nanoparticles

**Figure 2:**
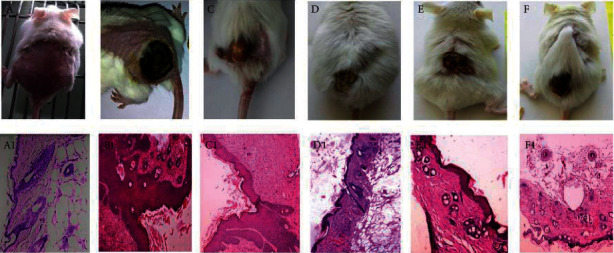
Representative images of the gross morphological appearance of mice skin tumors (a) untreated (nontumor control); (b) after 17 weeks of subcutaneous DMBA injection (untreated control); (c) after GNPs treatment; (d) after nisin-GNPs treatment; (e) after Dox-GNPs treatment; (f) after nisin-Dox-GNPs treatment for 7 weeks after skin tumor induction. Photomicrographs of histopathology that correspond (a1–f1).

Gold nanoparticles are among the most widely used types of nanoparticles for treatment due to their astounding properties in the treatment, diagnosis, and evaluation of skin infection, including skin growth [38]. Preet et al. demonstrated the creation of gold nanoparticles in nisin packaged with doxorubicin for therapeutic effects on murine skin. According to related reviews, Au nanoparticles aided the distribution of nisin doxorubicin in malignant growth cells. DMBA cells in laboratory cells have been refined. When measured at the end of treatment, the results show a significant decrease in growth volume. Furthermore, there is an expected decrease in serum levels of all cytokines tested for NF-, TNF-, TNF-, and IL-10, as well as a possible increase in the levels of oxygen responses in tissues and lipid peroxides [39]. Nirmala et al. advocated for Au nanoparticles to be used in apoptosis and to prevent harmful adherence to basic inhibitory remodeling. The cytotoxicity of gold nanoparticles may combine with the phenolic compounds of *V. vinifera* compounds, as well as the complexity of structured Au nanoparticles, causing decay and cell growth, as shown in Figure 2 [40].

Fratoddi et al. stimulate active gold nanoparticles modeled with methotrexate to detect serious irritants. Blood tests did not show any significant differences in platelet count and ALT and AST levels at baseline and at the end of treatment. MTX was used to package the efficacy of Au nanoparticles for gold nanoparticles in psoriasis rat models, which were tested in vitro and in vivo. A mouse model of psoriasis can be improved by administering gold nanoparticle treatment to stimulate epidermal hardening, keratinocyte proliferation, and provocative penetration. The K6CD3, Ki67, and CD8 used to test immunochemistry showed a decrease in spots in the AuNP in terms of cell proliferation, irritation, and epidermal firmness [41].

### 2.4. Iron Oxide Nanoparticles

In the pharmaceutical industry, the manufacturing of drugs with high specificity is rather difficult. Therefore, the iron oxide nanoparticles were used to create the superparamagnetic iron oxide nanoparticles to enhance the drug interaction with the receiver and to increase the abilities of transmission. Furthermore, drug concentrations can be controlled due to high effectivity and efficiency [42]. Rao et al. stimulated EPI-SPION to correct skin carcinoma through a transdermal course. The modification of SPION brings a medical vehicle that used the attraction of chemotherapy for dermatitis. Reviews have shown the ability of iron oxide nanoparticles to transmit simple transdermal signals to melanoma skin. Examination of cell lines for malignant skin growth in WM266 cells and HaCaT keratinocyte showed that SPION has significant similarities. In vitro testing focuses on testing whether the SPION stacked EPI can saturate the skin [43]. Cengelli et al. focus on the interaction of biocompatible cationic amino tiny superparamagnetic iron oxide nanoparticles with human cells in various cultures using electron microscopy and biological chemical processes. The results showed the secretion of amino-SPIONs by human melanoma cells. The mechanism of action of clathrin is altered and limited to the lysosome, which begins to stimulate and reduce the release of cathepsin D and transferrin receptors into skin fibroblasts [44]. Reduced fitness and damage were found in cell lines treated by FeO nanoparticles. The results have shown that iron oxide nanoparticles can enhance the corrective effect with low correction which may be a powerful and effective treatment for skin diseases; furthermore, the authors focused on photon-initiated treatment of skin melanoma and the potentially acute effect of iron oxide nanoparticles. X-beams photon therapy was evaluated with a focus on mice with CT26 growth [45]. Musazi et al. focused on the photon-initiated treatment of skin melanoma and the potentially acute effect of iron oxide nanoparticles. The results have shown that iron oxide nanoparticles can enhance the corrective effect with low correction which may be a potent and effective treatment for fatal skin diseases. X-beams photon therapy was evaluated with a focus on mice with CT26 growth. Reduced fitness and damage were found in cell lines treated by FeO nanoparticles [46].

### 2.5. Polymeric Nanoparticles

**Figure 3:**
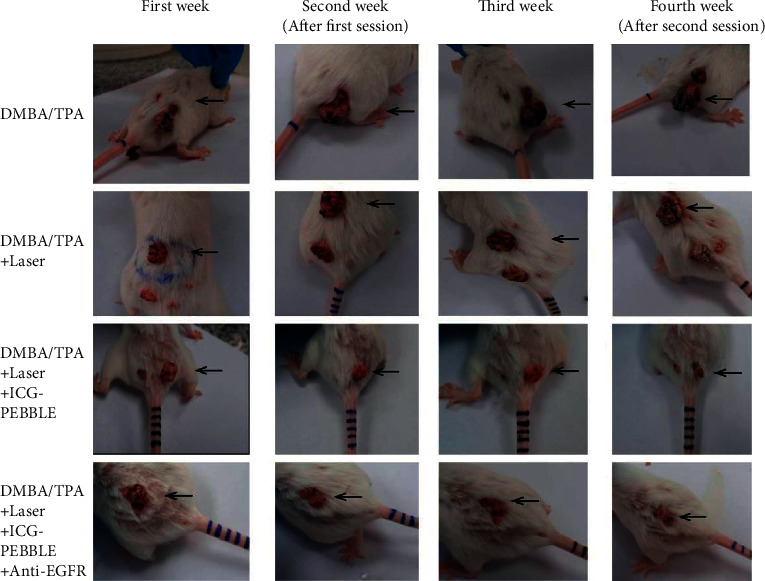
Photographs of tumor-bearing mice and tumor progression during PDT four-week experiments from various organizations.

One of the most significant nanoparticles for the skin is polymeric nanoparticles. Polymeric nanoparticles are of extraordinary importance to skin use due to their superior protection and controlled delivery and fluid retention by a polymeric grid to penetrate the skin [47]. Higher doses of the proteins and peptides described in recent years include immune disorders, diseases, and other complications of growth medicine. The epitome of the hair development compound in the PLGA nanoparticles will be enhanced by its full inner lining up to twice as a result of the control arrangement [47]. Chitosan, a deacetylated compound of chitin, is a perishable polymer containing glucosamine units. Over the microbial counter, oxidant toxic properties, and cooling properties make chitosan an ideal vehicle for medical attention to treat skin problems. In any case, at normal pH, the accumulation of chitosan amine is protonated, and in this way, chitosan is charged at a fixed rate. The cationic charge may be used by nanoparticles systematically in conjunction with polyanions to effectively synthesize anionic drugs through electrostatic interactions and to enhance cell secretion of epitomized chitosan nanoparticles [48]. Nair et al. showed that human similarity of acyclovir nanoparticles led to improved drug penetration, lowering of images, and increased drug exposure in pigskin [49]. Özbaş-Turan et al. showed that antisense oligonucleotide-stacked chitosan nanoparticles at 15–90 *μ*g showed significant inhibition of *β*-lady expression following 6 days of mouse transmission [50]. An expert in counter-neoplastic surgery, Dias, and his colleagues show that imiquimod is used to treat dermatitis, but the significant number of adverse effects near the base discovered through the use of a small penetration skin may interfere with the condition's ability to function properly. The corresponding review is aimed at assessing the antagonistic antigenic effect and resistance of the plant to the polymeric nanoparticles produced by Imq. The antagonistic antigenic content of the developed system was tested in chicken chorioallantoic incipient organisms. Its chemopreventive ability has also been tested in a mouse carcinogenesis model. A planned transport framework may be used as an alternative to treating diseases associated with vessel planning, as well as to improve skin function and the introduction of solvents to treat skin diseases such as melanoma of the skin [51]. Gamal-Eldeen et al. attempted to match the green color of indocyanine to polymeric nanoparticles in order to improve the water solubility of stagnant ICG components as shown in Figure 3. This study assessed the suitability of PDT resistance for EGFR form and frees ICG-PEEBBLE. Corresponding findings clearly showed that COX-2, TNF-*α*, i.e., growth corruption factor, and 5-LOX, which is 5-lipoxygenase decreased. In addition, apoptosis, caspase-3, and histone acetylation were induced in postmenopausal mice after PDT using these two types of definitions; nevertheless, a closed review corresponds to the formation of indocyanine green [52].

Pandey et al. focused on atopic dermatitis while producing chitosan nanoparticles of dermal concentrated and enlarged subcutaneous tissue. The nanoparticles created were studied with a record of polydispersity, zeta strength, molecular size, morphology, etc. In vitro discharge focuses on exposing the underlying skin regeneration. The entire BMV study was largely based on BMV-CS nanoparticles; however, the amount of drugs in the epidermis and dermis was significantly higher for HA-BMVCS nanoparticles, compared to BMV-CS nanoparticles; additionally, it has been proposed that HABMV-CS is a productive transfer platform for additional specific transfers and an enhanced enemy of promotional adequacy [53]. Nanoparticles were created by Bhatnagar et al. to improve chemotherapy production against skin melanoma in small parts. The author promoted bromelain epitomized poly (lactic-co-glycolic corrosive) and focused on counter cancer effects on the mouse model of skin tumorigenesis. The accompanying review results revealed an enlarged enemy of the neoplastic effect of nanoparticles in a phase II skin disease model. Decreased cell growth, decreased tumorigenesis percentage, and rat mortality rate were found in BL-infected nanoparticles compared to free BL [54].

Bayat et al. considered bromelain which is an example of chitosan nanofibers for edible lesions in experimental creature models. Chitosan nanofiber packed with bromelain was created by electrospinning strategy. The physiochemical properties of nanofibers were investigated. Cytotoxicity tests were also performed using Alamar blue. For 21 days, rats were used to study the chitosan nanofiber preparation process. Furthermore, the study showed that 2% of the chitosan w/v bromelain nanofiber model was effective in correcting skin reactions [55].

## 3. Nanomaterials for Skin Cancer Diagnosis

Once normal cells change and spread madly, harmful growth begins and causes cancer. Dangerous skin growth is diagnosed in 3 million Americans each year, making the disease the most dangerous type of growth known nationally and internationally [56]. Degenerative skin growth usually exhibits 4 types of diseases including squamous, basal, Merkel, and melanoma cell carcinoma [57]. Melanoma started with melanocytes and was considered a dangerous growth factor in the skin. There are a few evils in human strategy [58]. Biosensor or nanobiosensor science is commonly used to identify skin disease biomarkers. A nanosensor is a cognitive tool used in the physical evidence of a living thing. Nanomedicine plays a major role in the formation of nanosensors [59]. To detect dangerous skin growth, various electrochemical and optical biosensors have been developed, which will be discussed further below. It is frequently stated that the advancement of nanomedicine and its impact on the formation of highly sensitive metals is one of the most inspiring solutions to address a portion of the problem identified by the growing need for dramatic, rapid, and effective detoxification methods for skin disease.

One of the fastest biomarker markers for melanoma is lactate dehydrogenase [60]. The biomarker identifier associated with harmful skin growth has provided new pieces of information in the field of theranostics [61]. There is another prognostic biomarker in patients with malignant skin metastatic serum S100B levels [62]. BRAF V600 may be the best biomarker for metastatic melanoma. Approval of tyrosinase mRNAs in serum or blood is another biomarker of metastatic melanoma [63]. In specific tests, microsomal prostaglandin E2 synthase-1 was implicated in melanoma or skin disease. In inflammatory cases, excessive exposure to cyclooxygenase-2 is a promising biomarker of cancer inflammogenesis, metastasis, and angiogenesis [64]. An important role in the movement of melanoma has been documented to be played by network metalloproteinases [65]. Many plants including malignant melanoma and calcium-blocking proteins S100 accumulate as S100B, S100A9, and S100A4 have attracted as much interest as early biomarkers [66]. There are times when it does not show up until it is too late. Melanoma, on the other hand, shows a steady growth rate of DNA [67]. Melanoma biomarkers may include microRNAs or long-lasting noncoded RNAs, such as RNAs [68]. miRNA19a, miRNA21, miRNA149, miRNA17, and miRNA126 were isolated from the exosomes of patients with metastatic melanoma [69]. In general, the disclosure of biomarkers detected in fluid biopsies is less rapid compared to genomics and genomics biomarkers. Among the variables that limit the use of suitable biomarkers in point of care and applications are the lack of repetition, development strategies, and cycle time. Prior to advertising, biomarkers must meet strict rules by management professionals. Similarly, many of the proposed biomarkers in a clinical application may not be legally satisfactory.

Dermoscopy, optical intelligence tomography, and ultrasonography are used to identify harmful skin growth [70]. Skin Self-Exam skin self-examination is a strategy to identify any repairs on the skin or in unexpected areas. SSE is a basic strategy for classifying skin disease by a 6-half predominance of self-analyzed skin malignant growth frequency [71]. In dermoscopy, optical amplification by 10 and liquid immersion were used to identify skin lesions as a noninvasive thought process. Records of the unique appearance of skin cells and their physical structure produce the final image difference in the existing image. In melanin and keratin, the largest difference has a record of 1.7 and 1.5, respectively. High-recurrence ultrasonography has taken high-resolution images to detect various types of harmful skin growth. This high frequency helps the frame visualize parts of the skin, epidermis and dermis, and vessels to the end of the skin disease. Compared with an unconfirmed eye examination, dermoscopy is less effective in diagnosing melanoma at about 20 and 10%, respectively. From mild to severe melanoma, dermoscopy describes the development of skin lesions [72]. In determining dermatitis, these common procedures have become more common, but important barriers have kept them from being used in the real world.

**Figure 4:**
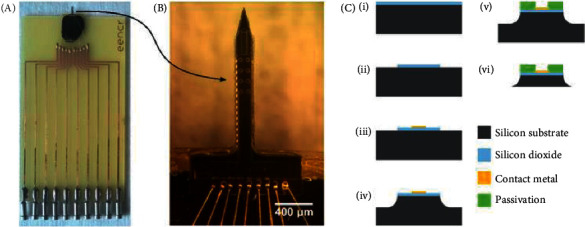
(a) Illustration of a packed electrochemical testing instrument. (b) Illustration of a needle-shaped electrode with 850 m gold disc electrodes. (c) Diagram of the fabrication method used for device production.

The vast majority of these methods require a successful professional to implement. In diagnosing skin disease, infrared light was used in optical cognizance tomography to make noninvasive communication. The presence of strong, hypoechogenic, and thick tissue raises the risk of dangerous skin growth during an ultrasound examination. OCT is appropriate in cases of misunderstood ultrasonography. But a cancerous lesion less than 1 mm in diameter is examined by OCT [73]. An effective and minimally invasive method of determining the cost of malignant growth of melanoma is the electrochemical ID of active biomarker melanoma, which completely improves sensitivity and transparency (Figure 4). Biosensors are usually tools used in the field of electrochemical biomarkers and can be used by speculation to construct a disease mark by a careful examination [74].

Immunosensors are effective in diagnosing diseases and are delivered using electrochemical transducer-moored antibodies. The use of nanoparticles in immunosensors enables natural macromolecules with high organic energy and movement to be able to move efficiently due to their high magnetic field and wide surface area [75]. A major component of electrochemical immunosensors is relied upon to combine reactive and stable components by incorporating stagnant biomolecules [76, 77]. Seenivasan et al. stimulated new immunosensors based on SiO_2_ NPs and polypyrrole nanocomposite to detect the destructive growth of melanoma [78]. The proposed immunosensor was particularly mild with 20/mL cell exposure, accurate and repetitive, and the Ab-prepared abstract terminal was no longer present for 14 days. The specific mechanism depends on the interaction between the MC1R immune response and the melanocortin one receptor antigen in the cell area. In another study, Ren et al. refer to a composite sheet of nanomesoporous Co_3_O_4_ i.e., Au/Co_3_O_4,_ and aminated graphene, i.e., GS-NH2 immunosensor based on Au NPs for melanoma attachment atom antigen ID [79]. Electrochemical or optical sensors can be used to look for body fluids, such as vomiting or sweating [80].

To quickly diagnose skin melanoma, Bianca et al. stimulated a flexible nanosensor and a small nonobstructive needle sensor. The wearable electrochemical sensors had the option to differentiate the presence of tyrosinase. The flexible sensor showed strong resistance to deformity, while the small empty needle unit was loaded with catechol-coated carbon glue to measure the lower TYR degrees in the muscle. Indeed, one-sided measurement was obtained by advertising the CAT substrate of the TYR chemical marker and amperometrically measuring BQ reaction at a low potential of 0.25 V. The resulting epidermal swath and wearable microneedle sensors demonstrated great scientific ingenuity and provided strong evidence for testing TYR biomarkers on both the skin surface and inside the skin [81].

The EIS framework is often reversed to diagnose skin disease. It incorporates an anode extraction test that distinguishes between abnormal and normal skin lesions using electrical impendence types. Electrochemical cell-substrate impedance detecting and electrochemical impedance spectroscopy are commonly used techniques to show individual cells based on a specific frequency of cellular responses and long-term physiological cell responses [82]. Consequently, Prathap et al. have preserved a very weak electrochemical impedance spectroscopy method, which is useful in the direct and rapid isolation of melanomas cells linked to MC1R PANI-NFs [83]. The current cell and the target had a good direct correlation. There are a number of advantages to using nanowires made of silicon to analyze visual evidence, including the ability to see changes at levels that are not possible with other apparatus [84]. In order to separate the two signals, both signal size and response power are used. They found separation and regular integration of TROY response with a neutralizer through their response models. Recognizing the symptoms of serum melanoma biomarkers, for example, a newly diagnosed TROY large family member is a low to high priority. Maedler et al. calculated the TROY ID in various focused support and silicon nanowires. The impact of nanosensors was assessed by comparing the signal with the findings in the support system [85].

Despite the benefits of using electrochemical methods, the harmful growth of the skin that separates the evidence in its early stages requires a concentration of the body fluid that lacks repair. In order to improve the recovery power and accuracy of the analysis, the combination of multimodal images and editing elements within the nanoplatform is essential. Combining electrochemical methods with late structured electrodynamic structures and electrochemical detecting nanoparticles can also increase the barriers to detection and vulnerability in new biomarkers. Significant advances in nanoscience, bioengineering, subatomic science, and computer science are relied upon to open the way to bring in accurate electrochemical biosensors to improve modified treatment systems for specific diseases.

Like early-stage biosensors, light processes have received surprising attention since late [86]. As a manifestation of skin diseases, including melanoma, extended tyrosinase actuation is a marker. In melanin biosynthesis, tyrosinase is an important biomarker for the detection of actinic damage, vitiligo, and melanoma [87]. Hu et al. conducted basic fluorescent tests based on carbon quantum spots associated with dopamine for TYR implementation [88]. In a separate study, Li et al. promoted oligonucleotide fluorescent experiments with pyrene mutations and in situ mix of nucleoside phosphoramidites and conversion of pyrene phosphorus to the internal phosphate core [89]. Fluorescent markers have been used to treat chronic melanoma as a logical thinking tool. They showed that the analysis of well-adjusted analysts made fluorescence strength increase rapidly and basically up to 23.5 times, although analysts with a single bungle on both sides of phosphate-connected pyrene showed a flexible fluorescence. In another review, Sweeny et al. showed that expansion of antiangiogenic therapy promotes the retention of fluorescent-marked monoclonal antibodies within melanoma cancer [90]. High-impact photoelectrochemical nanosensor was calculated by Erhu et al. to recognize DNA. The nanosensor relies on the development of a study signal, hybridization chain response, synergist component collection, and basic phosphatase. One of the most popular new experiments that have attracted a lot of interest from analysts due to its cost and high impact is the photoelectrochemical biosensing strategy [91, 92]. The recommended PEC biosensor has shown excellent performance with amazing sensitivity. In another study, T-DNA was separated by a LOD of 0.052 fM and a direct reaction scope of 0.1 fM to 100 pM. Lopes et al. showed close infrared input at 750 to 1400 nm. Covered Au NPs also show a circular shape with a normal range across 297 nm without any toxic effect on yeast and tested cell lines. In any case, the function of the B16F10 cell line was reduced by 20 percent after laser implantation. These reviews have shown promising results for melanoma treatment and determination [93].

## 4. Conclusions and Outlook Prospective

Nanodermatology is a new topic of study with potential medical uses. In modern society, nanoparticles have been widely used. It has been found that nanotechnology-enhanced inorganic nanomaterials and antibodies can detect a wide range of skin disorders and cancers. This has led to the development of newer versions.

Skin cancer detection is a fast-moving disease that requires careful consideration. Melanoma's mortality rate is reduced by conventional methods, but these methods are not suitable for use in the clinic and recent advances in nanosensors have led to new methods for detecting melanoma in clinical trials.

Point of Care instruments must be used to implement detection strategies in the clinical setting. It is the lack of perfect biomarkers that is the most significant challenge to electrochemical melanoma detection. Low selectivity in several biomarkers raises the risk of incorrect results.

Electrochemical biosensors and electro-active substances can be combined with electrochemical procedures to increase the sensitivity and detection limits of newer biomarkers. As a result, nanomaterials tend to combine bioactivity, permeability, and catalytic potential to enhance the final signal, which is why they are so popular. There are going to be a lot more electrochemical biosensors in the near future because of new developments in genetic engineering, computational biology, and molecular biology. It is also critical to use nanotechnology to create new electrospun fibers that could be used as drug delivery mats for melanoma treatment. We recommend further research into these agents to determine the possibility of developing topical drug delivery systems via microemulsion or scaffolds for topical application. Only in this manner can we target the tumor and design drug-loaded nanofibers or nanoparticles, which may yield the best results in clinical melanoma therapy.

Nanomaterials allow for more efficient use of therapies such as immunotherapies and gene therapies, which is important given their high cost. The versatility of these nanocarriers grows exponentially because while their use increases the cost of therapy, it comes with innumerable benefits that, in terms of cost-effectiveness, are worth admitting, as these benefits lead to better chances for diagnosis and treatment, as well as allowing the use of theranostics in cancer patients. The use of nanomaterials, like other strategies, could make both types of therapies clinically viable. Although the nanotechnology field offers several cancer-fighting strategies, many more efforts are required to develop effective and safe therapies.  Table 1 presents the advantages and disadvantages of various types of nanoparticles used in skin cancer combat.

## Figures and Tables

**Table 1:** Advantages and disadvantages of various types of nanoparticles used in skin cancer combat.

Nanomaterials	Advantages	Disadvantages
Lipid nanoparticles	High biocompatibility	Possibility of drug expulsion
	Limited side effects	
	Enhanced skin perforation	

Carbon nanotubes	High stability	Lack of solubility in aqueous media
	High cytoprotective properties strong impact on oxidants	

Gold nanoparticles	Large surface area	Toxicity effects
	High sensitivity and selectivity	

Iron oxide nanoparticles	Great drug encapsulating capacity	Low saturation magnetization
	Great targeted delivery efficiency	

Polymeric nanoparticles	Great stability	Toxic degradation residual
	Controlled drug release	

Electrochemical nanosensors	High biological stability	Limited shelf life
	High adsorption ability	
	Wide surface area	

## Data Availability

The data used to support the findings of this study are included within the article.
